# Studies on the purification of polypeptide from sika antler plate and activities of antitumor

**DOI:** 10.1186/s12906-015-0845-7

**Published:** 2015-09-18

**Authors:** Wei Hu, Lin Qi, Yu H. Tian, Rui Hu, Lei Wu, Xing Y. Meng

**Affiliations:** College of Life Sciences, Jilin Agriculture University, No. 2888 Xincheng Road, Changchun, Jilin province 130118 China; China Agriculture University, Beijing, 100094 China; The branch institute of Liaodong college agronomy courtyard, Dandong, 118001 China

**Keywords:** Sika, Antler plate, Monomer polypeptide, Antitumor activities, Cell proliferation

## Abstract

**Background:**

We isolated a novel monomeric peptide from antler plate polypeptide (APP) of sika deer and found that it inhibited rat breast cancer cell proliferation and telomerase activity.

**Methods:**

The molecular mass and purity of this polypeptide was determined by ultra performance liquid chromatography (UPLC) and Bruker micOTOF OllQ TOF mass spectrometry, respectively. The full amino-acid sequence of the monomeric peptide was analyzed by sequential Edman degradation using a protein/peptide sequencer. The APP-1 markedly inhibited rat breast cancer cell proliferation as determined with an 3-(4,5-dimethyl-2-thiazolyl)-2,5-diphenyl-2-H- tetrazolium bromide (MTT) assay. Then, we used flow cytometry to detect the effects of the monomeric peptide on cell cycle. Relative quantitative fluorescence PCR was used to analyze the expression level telomerase reverse transcriptase (TERT).

**Results:**

The molecular mass and purity of this polypeptide was 10646 Da and 91.2 %. Amino acid sequence analyses indicated that the N-terminal amino-acid sequence of this monomeric peptide was: MTKLE DYLEG IVNIF HQYSV. The results showed that monomeric peptide halted most cancer cells stagnating in the G0/G1 phase. The percentage of cells in the G_0_/G_1_ is higher than control group after the monomeric peptide treatment. Relative quantitative fluorescence PCR results showed that TERT gene expression level obviously decreased after treatment with the monomeric peptide compared with control group.

**Conclusions:**

Collectively, the results suggest that this novel and monomeric APP has antitumor activities and imply that it is likely an important component of antitumor activities in antler plate polypeptide.

## Background

Although velvet antler has been used clinically for thousands of years in China, the antler plate (AP) from *Cervus Nippon Temminck* was not previously used in traditional Chinese medicine. An antler plate (AP) is the ossified disc between the front of the skull and the velvet antler of sika deer [1]. AP especially from sika deer produced in Jilin Province is a precious Chinese medicine material. A characteristic use of AP is to treat mastitis [2–4]. However, it is so rigid that it is difficult to shatter. For this reason, researchers have not yet identified the active components. Here, we report a standardized procedure to overcome this problem and demonstrate that it can help in utilizing AP.

Velvet antlers, which contain cartilage, bone, epidermis, blood vessels, nerves, and other tissues, can grow rapidly [5, 6]. Scientists in New Zealand have demonstrated that there are a number of growth factors and their receptors in velvet antler tip and hypothesize that these underlie the observed rapid growth [7, 8]. AP is the ossified disc of velvet antler, and it has exerts some of the same activities as velvet antler, including promoting wound healing and anti-inflammatory and anti-bacterial effects [1].

The content of protein in antler plate can reaches up to 40 %, being one of the main functional components in antler plate. However, the larger the molecular mass of protein, the worse the absorption extent. The monomeric protein polypeptide is much easier to be absorbed by the organism compared with the protein of larger molecular mass. Then, on the basis of former researches of antler plate, this study aim to found the active component that of true use, choose breast cancer cell line MA782 in mice as research object *in vitro*, and discuss the effect of antler plate on breast cancer cell line MA782 in mice, which can provide scientific basis to the further development of antler plate as anticancer resources and also provide possibility to the effective control of the breast cancer.

In recent years, more and more scientists are focusing on small molecular protein monomers during the research of antler plate. These studies showed that AP contains a variety of different active ingredients that could cure breast hyperplasia and inhibit of breast cancer [9–11]. Huang et al previously reported the isolation of an active polypeptide from AP of sika deer with a molecular mass of 7127.6u [12], and further demonstrated that this polypeptide exerted a glucose-cutting activity. We were interested in determining if other polypeptides with useful biological activities could be derived from AP. In this paper, we purified a novel antler plate polypeptide (APP, 10646 Da) from the AP of sika deer using column chromatography and determined that it could inhibit the proliferation and telomerase activity of rat breast cancer cell line MA782.

## Methods

### Cell line

MA782 cells are rat breast cancer cells, growing sticked a wall. Its cell form is epithelioid. MA782 cells were purchased from (China Center for Type Culture Collection, Wuhan, China) and maintained in RPMI 1640 with 10 % fetal bovine serum (Gibco/Invitrogen, Carlsbad, CA, USA) and 1 % antibiotics (penicillin and streptomycin) (Gibco/Invitrogen).

### Isolation and extraction of total polypeptide

This study was approved by the ethics committee of the Institute of Life Sciences, Jilin Agriculture University, Changchun, China. Samples of hard antler plate were obtained from a 3 year-old male *Cervus nippon* provided by Jilin Agricultural University Deer Farm (Changchun, China). The raw material (500 g) was extracted with precooled acetic acid solution and further concentrated, and the residue was lyophilized. The resulting freeze-dried material (500 mg) was demineralized with a SephadexG-25 column (10 × 1000 mm) (Pharmacia, China) that had been pre-equili brated with deionized water. Proteins peaks were collected and lyophilized.

### Monomer peptide separation and purification

As described above, the total peptide (300 mg dry mass) was applied to a Sephadex G-50 column (10 × 1000 mm) that had been pre-equilibrated with distilled water. The total peptide (S2) markedly inhibited MA782 cell proliferation after 72 h (in doses from 10–30 mg/L, as determined with MTT assay). Lyophilized polypeptide S2 was then purified using reverse-phase high-performance liquid chromatography (HPLC, Zorbax 300SB C18 9.4 × 250 mm) (China) [13]. Elution was performed with a linear gradient system from solvent A (0.1 % trifluoroacetic acid in 15 % acetonitrile) to solvent B (0.085 % trifluoroacetic acid in 70 % acetonitrile) at a flow rate of 1 mL/min over 30 min, and absorbance was detected at 280 nm. The final yield of Peptide samples (APPs) was 5.0 mg (0.010 %, w/w, AP).

### Analysis and identification of the monomer peptide

Peptide samples were hydrolyzed at 110 °C for 24 h with 6 M HCl containing 0.1 % phenol, and the hydrolyzate was analyzed with a Beckman model 6300 automatic amino acid analyzer (Beckman Coulter, Brea, CA, USA). Purity and molecular mass was determined by the UPLC-MS/MS system. The UPLC-MS/MS system consisted of an Agilent 1290 system (Aglient Technologies, USA) and a Bruker micrOTOF QII (Bruker Daltonics, USA) mass spectrometer equipped with an electrospray ionization (ESI) source. Chromatographic separation was achieved on a Zorbax Stable Bond C18 300A analytical column (4.6 × 250 mm, 5 μm; Aglient Technologies, USA) operated at 30 °C. The mobile phase consisted of 0.1 % TFA water (A) and acetonitrile (B), formed by a gradient elution of 5–5 % (v/v) B at 0–5 min, 5–80 % B at 5–55 min, and 80–98 % B at 55–60 min. The flow rate was 1.0 mL/min. The mass spectrometer was operated in the positive ionization mode. Optimized MS parameters of capillary and endplate voltage were 4.5 kV and 500 V, respectively. Nitrogen was used as the dry gas and nebulizer gas at a rate of 6.0 L/min and pressure 0.8 bar. The dry gas temperature was set at 200 °C. Argon was used as the collision gas and collision energy was set to 8 ev for all of the transitions. Data were acquired and analyzed with Bruker Data Analysis (version 4.0 SP 1) [14]. Amino-acid sequence was determined by sequential Edman degradation using a protein/peptide sequencer at the Beijing Analyse Centre of Biomedicine (China). All the processes were performed automatically.

### MTT assay

Prepare the protein solution of which the concentration is 20 μg/mL from the purified monomeric peptide freeze dried using RPMI-1640 culture solution that do not contain serum, and then filtration sterilization. Then take cells in logarithmic phase, digest them by trypsin, prepare single cell suspension using RPMI-1640 complete culture solution, inoculate them into the culture plate of 96 holes in accordance with the density of 3 × 10^3^/hole after cell counting, the volume of each hole is 200 μL. Move the culture plate into CO_2_ incubator, culture it for 24 h under the condition of 37 °C, 5 %CO_2_, discard the culture solution by suction after cell attachment. Take cells that have not accepted the monomeric protein processing as control group, and cells that have accepted the monomeric protein processing as experimental group. The concentration of the protein is 20 μg/mL respectively, and incubation time is 24 h, 48 h, 72 h respectively. Add 20 μL MTT solution to each hole at 4 h before the end of the incubation. Abandon the supernatant at the end of the incubation, add 200 μL DMSO to each hole, place the plate in incubator for 30 min to let the crystal substance in cells dissolve sufficiently. Choose the wavelength of 490 nm, and measure the light absorption value of each hole using ELIASA [15].

### Cell cycle analysis

Cells were seeded in 6-well plates at 50 % confluency. The next day, we added the monomeric peptide (20 μg/mL final concentration). After 24, 48, and 72 h, cells were trypsinized, washed with phosphate-buffered saline (PBS), and fixed in cold ethanol (75 %) for 24 h at 4 °C. Cells were then washed with PBS and resuspended in propidium iodium (20 mg/ml) solution containing RNAse (200 mg/ml) for 30 min in a dark room. Cellular DNA content was determined with a flow cytometer (Becton Dickinson, Franklin Lakes, NJ, USA) [15].

### TERT mRNA level detection

A TaqMan RNA Assay (Invitrogen) was used for TERT quantification. The monomeric peptide (20 μg/mL final concentration) was transfected into rat breast cancer cells that reached a growth density of 80 %, and total RNA was extracted from cell culture pellets using TRIzol reagent (Invitrogen) after 24, 48, and 72 h. For reverse transcription, 1 mg of RNA was used and retranscripted with a SuperScript II reverse transcriptase kit (Invitrogen). The resulting cDNA was amplified with TaqMan in the presence of 1.5 μl gene-specific primers, 1 μl probes, and TaqMan Master Mix. The primers and probes were supplied by Applied Biosystems (Foster City, CA, USA) and the sequences are listed in Table 1. Quantitative real-time PCR was carried out using Applied Biosystems Step One Plus Real-Time PCP (Shanghai CpG Biotech Co., Ltd., CA). TERT mRNA abundance was calculated as the ratio of the copy number of target mRNA normalized to the copy number of β-actin mRNA. Reactions were run in duplicates and repeated at least three times in optical 96-well plates. Finally, we calculated the corresponding level using the 2^-△△Ct^ = 2^-△Ct treatment -△Ct reference^ method. Each real-time PCR assay was performed at least three times [16].

**Table 1:** Quantitative real-time PCR primers and probes

TERT	Tert -Forward:5- CAACAGAAAGTCATCAACAAAACG–3
	Tert -Reverse:5- GCTCCAGCCTATCCACCCT–3
	Tert -Taqman-MGB-Probe:5- AAGCAACCCATCCCG–3
β-actin (mBA)	mBA-MGB-Forward: GACAGGATGCAGAAGGAGATTACTG
	mBA-MGB-Reverse: GCTGATCCACATCTGCTGGAA
	mBA-MGB-FAM: FAGATCAAGATCATTGCTCCP

### Data analysis

All data are presented as means ± standard deviation (SD). SPSS version 12.0 software (SPSS Inc., Chicago, IL, USA) was used for all statistical analyses, and *p*-values <0.05 were considered to be statistically significant.

## Results

### Separation and purification of a new monomeric peptide from APP

Crude total polypeptide extract was treated with acetic acid buffer and ethanol, analyzed by SDS-PAGE, and the Coomassie brilliant blue-stained gel (a single band of approx.3 kDa) suggested that it contained five polypeptides. Final purification of crude total peptide extract was accomplished by column chromato- graphy on a Sephadex G-50 and reverse-phase HPLC.

**Fig. 1:**
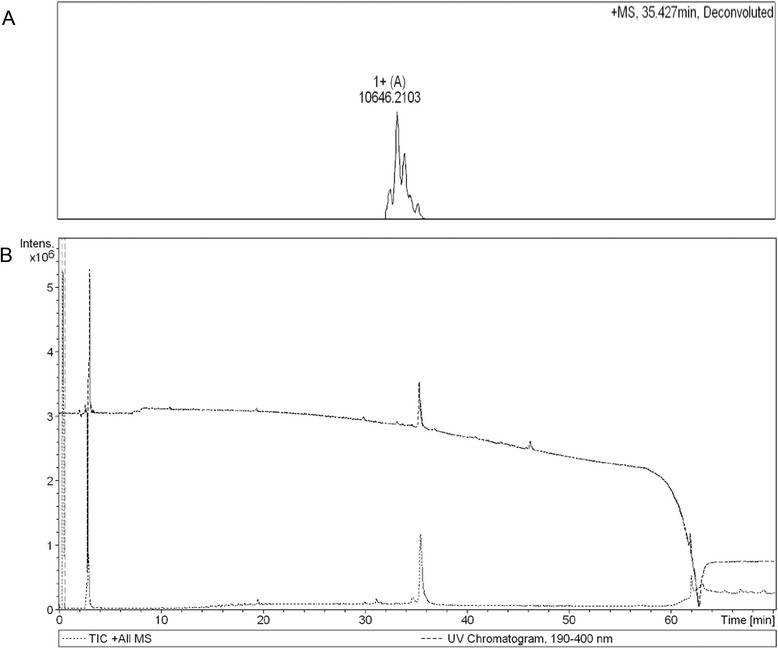
Purity identification and molecular mass detection of the monomeric peptide. **a** Mass of the monomeric peptide was determined by Bruker micOTOF OllQ TOF with an experimental accuracy of ± 0.1 %, the peak is due to solvent interference. The molecular mass of this novel polypeptide was 10646 Da. **b** Purity identification of the monomeric peptide by utilizing the HPLC, The purity of this novel polypeptide was 91.2 %

APP was isolated as an amorphous white solid. Acid hydrolysis demonstrated that APP is composed of amino acids; alanine, glycine, glutamic acid, and proline were the main amino acid residues, and cysteine was not present (Table 2). The molecular mass determined by Bruker micOTOF OllQ TOF was about 10,646 Da. The purity of the purified peptide was demonstrated to be 91.2 % *via* HPLC (Fig. 1). An attempt to sequence the monomeric peptide directly *via* Edman degradation led to the conclusion that its N-terminus was open. Thus, the monomeric peptide was established as a linear polypeptide with the amino-acid sequence illustrated. The amino acid sequence of this monomeric peptide was MTKLEDYLEG IVNIFHQYSV. It was named APP-1.

**Table 2:** Amino acid composition in APP

Types of amino acid	Molar ratio of amino acid
Pro (P)	4.85
Gly (G)	3.42
Ala (A)	3.18
Leu (L)	1.34
Met (M)	0.36
Val (V)	1.12
Phe (F)	0.88
His (H)	0.39
Tyr (Y)	0.14
Asp (D)	1.85
Ser (S)	1.22
Arg (R)	2.70
Ile (I)	0.57
Thr (T)	0.84
Glu (E)	3.48
Lys (K)	1.49

### Restoration of APP-1 inhibits rat breast cancer cell growth

**Fig. 2:**
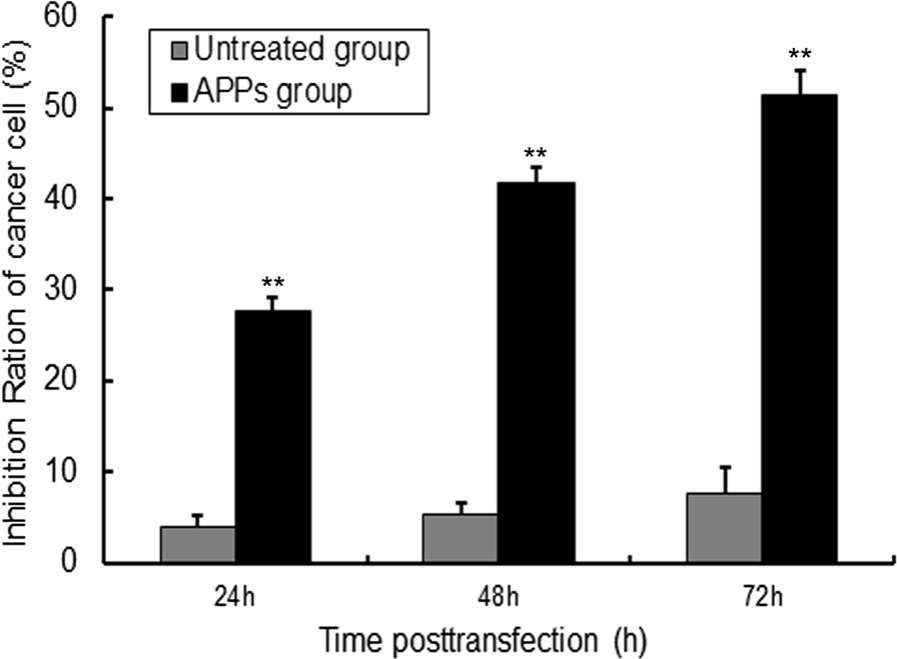
Effects of the novel monomeric peptide on MA782 cell proliferation using the MTT method. MA782 cells were treated with the monomeric peptide as indicated. The rate of cell inhibition was measured at the indicated times post-transfection using an MTT assay. Compared with the control, cell inhibition decreased when cells were treated with the monomeric peptide after 24 h, 48 h, and 72 h. Data are presented as the mean ± SD (*n* = 3). Similar results were obtained in three independent experiments. *P < 0.05; **P < 0.01, compared with the control

Rat breast cancer cell line MA782 was treated with the monomeric peptide (20 μg/mL) for 24, 48, and 72 h, and the effects of this monomeric peptide on cell proliferation were investigated using MTT assays. Compared with the control group, the proliferation of MA782 cells exhibited significantly decreased at 48 h after the monomeric peptide treatment (*P* < 0.01, Fig. 2).

### Effect of APP-1 on cell cycle

**Fig. 3:**
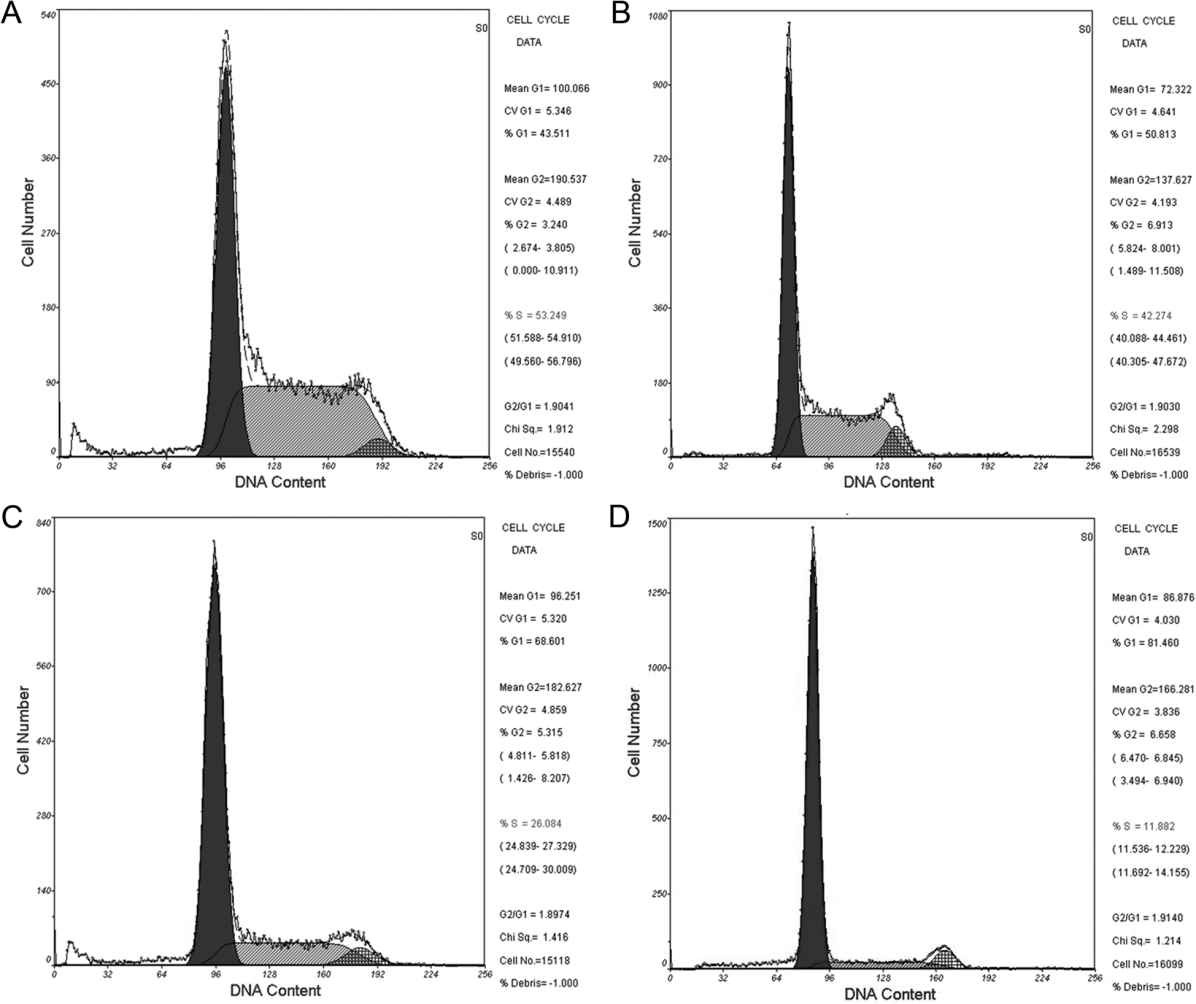
Cell cycle results before and after the monomeric peptide treatment for 24, 48, and 72 h, respectively (**b**, **c**, **d**). The figure **a** is the contral group. The y-axis indicates cell number, and the x-axis shows DNA content. The monomeric peptide prevented the entry of MA782 cells into S phase, and the percentages of cells in G_1_/G_2_ in the treated groups are higher than control group; this was especially pronounced in the 72 h treatment group

Rat breast cancer cell line MA782 was treated with the monomeric peptide (20 μg/mL) for 24, 48, and 72 h, and we used flow cytometry to assess cell cycle. The results showed that the monomeric peptide altered the percentage of cells in every phase. There was a clear decrease and increase in the number of cells in the S and G_0_/G_1_ phases, respectively. Over time, these differences became more apparent. Based on these results, we could infer that the monomeric peptide arrested the cell cycle, and most cells were in the G_0_/G_1_ phase (Fig. 3).

### Effect of APP-1 on TERT gene expression

**Fig. 4:**
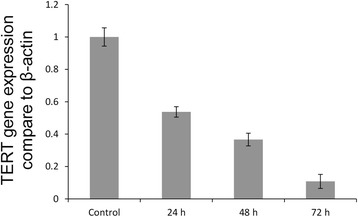
Detection of TERT gene expression level by real-time PCR. Rat MA782 cells were treated with the monomeric peptide, and TERT mRNA expression was assessed using quantitative PCR after 24, 48, and 72 h. Each experiment was performed in triplicate. TERT mRNA expression levels were significantly decreased at all-time points in comparison with control cells. Data are presented as the mean ± SD (*n* = 3). Similar results were obtained in three independent experiments. *P < 0.05; **P < 0.01, compared with the control

After treatment for 24, 48, and 72 h, we used TRIzol solution to extract total RNA from rat breast cancer cells. And 1 % agarose gel electrophoresis results showed that we isolated good-quality RNA. The three electrophoresis lanes for rRNA (28S, 18S, and 5S) were clear and complete without degradation or genomic DNA. Thus, total RNA was used as templates for reverse transcriptase and subsequent TERT and β-actin gene amplification. Relative fluorescence quantitative PCR showed that cells treated with the monomeric peptide (20 μg/mL) for 24, 48, and 72 h had decreased TERT gene levels compared with the control group (Fig. 4).

## Discussion

Gel chromatography and HPLC are widely used in peptide purification because they offer good separation, speed, high sample capacity, and high efficiency [17, 18]. The total peptide of AP contains five kinds of peptides according to SDS-PAGE analysis. We first used gel chromatography to separate them. Peptides with significantly different molecular weights are found in total peptide, but the Sephadex G-50 classification range is 1500–30,000 and could meet the requirements, the selection of Sephadex G-50 as the fill medium was appropriate. Our results show that this equipment could separate the peptide effectively, which offered a good foundation for the next step. We selected a more suitable method to determine the chromatography parameters and separated out a monomer peptide with 91.2 % purity. *Huang et al* [12] reported that the sika antler peptide isolated by ion exchange, gel filtration, and reverse phase chromatography, and MALDI-TOF-MS revealed that the molecular mass was 7127.6. This result was in accordance with ours, even though we used a much simpler purification process that saved time and significantly reduced sample loss. Peptide purification was good, and the different polarity of HPLC allowed us to separate the constituents and obtain high-purity APPs.

Native polypeptide drugs have low molecular weights, are easy to absorb, and have few side effects. They can directly correct cytokine imbalances on molecular and cellular levels. *Yunlu et al* [19] reported that they successfully isolated and purified a cytotoxin from Naja naja atra venom using gel filtration, ion-exchange column chromatography, and hydrophobic interaction chromatography. They performed *in vitro* experiments and demonstrated that this cytotoxin could inhibit proliferation in human hepatocellular carcinoma BEL-7404, human lung adenocarcinoma A549, and mouse melanoma B16 cells. *Huang et al* [12] separated a monomeric peptide with a molecular weight of 7127.6 and treated insulin-resistant HepG2 cells to determine if there were any effects on glucose consumption. The results showed that the monomeric polypeptide had obvious hypoglycemic activity.

We used the rat breast cancer cell line MA782 to assess the effects of APPs. Many studies have reported the sika AP can cure mastitis by inhibiting mammary gland hyperplasia. However, these treatments were comprised of many AP peptide components and other Chinese crude drugs in compound preparations. However, no breast cancer research utilizing monomeric sika AP polypeptide has been reported. Here, we obtained a monomeric peptide with a molecular weight of 10,646, which is different than that obtained by Huang. MTT experiment results indicated the novel polypeptide could inhibit the proliferation of MA782 cell. This may be because the molecular of monomer peptide was small, structure was simple, and it was easy to digestion and absorption. So the physiological function and nutrition value could be more obviously. Therefore, in the follow-up tests, we would select the novel monomer peptide to continue experiment later, so we can continue to evaluate monomer peptide inhibition effect from other aspect.

The basic characteristic of a tumor is uncontrolled cell growth, including reduced apoptosis or increased proliferation. Cancer is largely a cell cycle disease, and cell cycle suppression is an important topic in cell biology and oncology research. Daowei and colleagues demonstrated that earthworm extract can successfully inhibit ECA-109 and HeLa cancer cell lines [20]; the authors hypothesized that the tumor cells were arrested in the G0/G1 period, which eventually led to cell death due to lack of proliferation. *Shuqin et al* [21] verified that active, low molecular weight anticancer peptides extracted from goat spleen could inhibit nml-231 breast cancer cell proliferation by halting the cell cycle. ACBP stopped cells in the G_1_ period and inhibited DNA synthesis. Compared with the control group, the number of G_0_/G_1_ phase cells was significantly higher, and the number of S phase was significantly lower in cells treated with the monomeric peptide. These results demonstrate that the monomeric peptide can effectively halt the cell cycle in a breast cancer cell line.

Telomerase activity and the occurrence and development of most malignant tumors are closely correlated. Telomerase activity is detectable in 90 % of malignant tumor tissues, including lung, breast, prostate, and other major cancers, as well as most leukemias and lymphomas. The regulation of telomerase activity is complex. Telomerase is highly regulated during cell development and differentiation. Telomerase is a potential diagnostic and prognostic marker in human tumors consisting of a specialized ribonucle oprotein polymerase composed of the RNA subunit, human telomerase RNA (hTR), and a catalytic protein component, human telomerase reverse transcriptase (hTERT), which can elongate telomeric DNA using its own RNA subunit as a template. hTR is ubiquitously and equivalently expressed in both normal and tumor tissues, but the hTERT subunit is selectively expressed in a small subset of normal cells, tumor tissues, and tumor-derived cell lines, indicating that hTERT is the rate-limiting component of telomerase activity.

TERT catalyzes telomere lengthening. In immortalized cells, TERT gene expression levels increase, whereas it is not expressed in normal cells [22, 23]. TERT expression is directly proportional to telomerase activity, and it plays an important role in telomerase activity regulation [24, 25]. *Shan et al* [26] reported that a venom polypeptide increased apoptosis in the human hepatic carcinoma cell line BEL-7402 and decreased telomerase activity. Similarly, *Guisheng et al* [27] found that treating human hepatic carcinoma Bel7404 cells with scorpion venom polypeptide effectively reduced telomerase activity. Collectively, these lines of evidence suggest that telomerase activity inhibition is a possible mechanism for halting cancer cell proliferation.

We found that the monomeric peptide effectively decreased TERT mRNA levels compared with the control group, which corresponded to the observed MTT results and cell cycle changes. Our findings suggest that APPs might inhibit breast cancer cell proliferation by regulating telomerase activity. Others researchs have implicated that the TERT gene in regulation of breast cancer telomerase activity. Thus, it is possible that APPs also regulates telomerase activity by degrading TERT gene mRNA level. Perhaps inhibition of telomerase activity is one of mechanisms by which APPs prevents breast cancer cells from proliferating. However, whether APPs was the only component that lowered TERT expression requires further study. Even so, the results of this experiment expand the theoretical basis for further study into the anti-tumor mechanisms of sika AP.

## Conclusion

In conclusion, whether there are other active components in sika antler plate depends on how the native material is purified. We used a two-step process and obtained a new monomeric peptide that effectively inhibited MA782 cell proliferation. The mechanisms may involve a reduction in TERT gene expression, and cell cycle arrest. However, whether this single peptide has other functions should be researched further.
